# Effects of Sterilization With Hydrogen Peroxide and Chlorine Dioxide on the Filtration Efficiency of N95, KN95, and Surgical Face Masks

**DOI:** 10.1001/jamanetworkopen.2020.12099

**Published:** 2020-06-15

**Authors:** Changjie Cai, Evan L. Floyd

**Affiliations:** 1 Department of Occupational and Environmental Health, University of Oklahoma Health Sciences Center, University of Oklahoma, Oklahoma City

## Abstract

This quality improvement study examines the effects of sterilization with hydrogen peroxide and chlorine dioxide on the filtration efficiency of N95, KN95, and surgical face masks.

## Introduction

Owing to the coronavirus disease 2019 pandemic, there is a global shortage of masks needed to protect health care personnel.^1,2^ The US Centers for Disease Control and Prevention has suggested the potential reuse of disposable respirators to conserve available supplies.^3^ A study by Viscusi et al^4^ evaluated various sterilization methods for reuse of N95 masks. Although surgical masks should not be used as a substitute for N95s owing to lower fit quality,^5^ a randomized clinical trail by Radonovich et al^6^ found that there was no significant difference in the incidence of laboratory-confirmed influenza among health care personnel who used N95s vs surgical face masks. The Centers for Disease Control and Prevention listed KN95 masks (the Chinese version of the N95) as suitable alternatives to N95s when N95s are not available. However, to our knowledge, there are no studies regarding the effects of sterilization on the filtration efficiencies of KN95s or surgical face masks. The goal of this quality improvement study was to test the feasibility of reusing KN95s and surgical masks.

## Methods

The University of Oklahoma institutional review board determined that this study was not human research and was exempt from informed consent. This study is reported following the Standards for Quality Improvement Reporting Excellence (SQUIRE) reporting guideline. This study was conducted from March 25 to April 7, 2020.

We compared sterilization by plasma vapor hydrogen peroxide (H_2_O_2_) and chlorine dioxide (ClO_2_) on the filtration efficiencies of 3 types of masks, N95s (model 1860; 3M), KN95s (Civilian Antivirus; Qingdao Sophti Health Technology), and surgical face masks (model 1541; Dukal). Experiments were conducted using the test chamber illustrated in eFigure 1 in the Supplement. A stable salt aerosol (eFigure 2 in the Supplement) was generated using a 3-jet Collison nebulizer (CH Technologies) and 2% sodium chloride solution in accordance with National Institute of Occupational Safety and Health procedure No. TEB-APR-STP-0059. A scanning mobility particle sizer (model 3936; TSI) was used to measure the particle number concentration from 16.8 nm to 514 nm. All masks were preconditioned in an incubator at 38 °C and 100% relative humidity for 12 hours. For each mask, 5 samples were tested with 4 upstream measurements and 4 downstream measurements. Acceptable pressure drop was defined as less than 35 mm or 1.38 inch water for inhalation. We calculated the mean and SD of filtration efficiency and pressure drop for each type mask.

## Results

**Figure 1.**
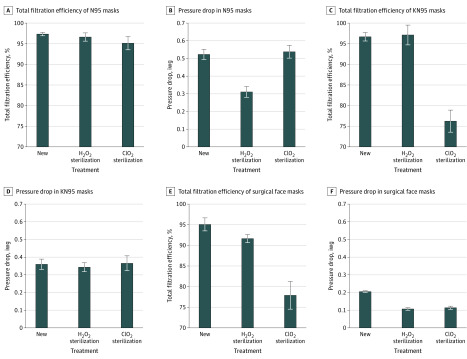
Effects of Sterilization on Overall Filtration Efficiency and Pressure Drop Error bars indicate SD; iwg, inches of water gauge; ClO_2_, chlorine dioxide; and H_2_O_2_, hydrogen peroxide.

The effects of sterilization on overall filtration efficiency and pressure drop are summarized in Figure 1. The mean (SD) filtration efficiencies of untreated masks were 97.3% (0.4%) for N95s, 96.7% (1.0%) for KN95s, and 95.1% (1.6%) for surgical face masks. After H_2_O_2_ sterilization, the filtration efficiencies were 96.6% (1.0%) for N95s, 97.1% (2.4%) for KN95s, and 91.6% (1.0%) for surgical face masks. The N95s and KN95s retained at least 95% efficiency, but the surgical face mask’s efficiency was reduced. After ClO_2_ sterilization, the filtration efficiencies were 95.1% (1.6%) for N95s, 76.2% (2.7%) for KN95s, and 77.9% (3.4%) for surgical face masks. The H_2_O_2_ treatment showed a small effect on the overall filtration efficiency of the tested masks, but the ClO_2_ treatment showed marked reduction in the overall filtration efficiency of the KN95s and surgical face masks. All pressure drop changes were within the acceptable range.

**Figure 2.**
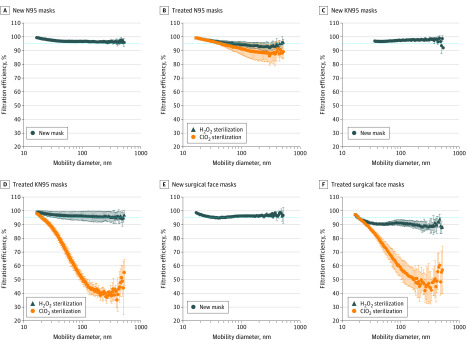
Effects of Sterilization on Filtration Efficiency by Aerosol Size Error bars indicate SD; ClO_2_, chlorine dioxide; and H_2_O_2_, hydrogen peroxide.

The effects of sterilization on filtration efficiency by aerosol size are presented in Figure 2. For all untreated masks, the filtration efficiencies by size were more than 95%. For N95s after ClO_2_ sterilization, the mean (SD) filtration efficiency for particles of approximately 300 nm decreased to approximately 86.2% (6.8%), although the overall filtration efficiency was retained at approximately 95%. Therefore, caution should be exercised when using this mask under this condition. The mean (SD) filtration efficiencies decreased to 40.8% (5.9%) for KN95s and 47.1% (14.4%) for surgical face masks for particles of approximately 300 nm.

## Discussion

This quality improvement study found that the sterilization processes had different effects on the filtration efficiencies of different masks. Sterilization with H_2_O_2_ had fewer negative effects than ClO_2_. In addition to considering the overall filtration efficiency, the filtration efficiency for particles similar to infectious agents should be considered. This study has some limitations, including the small variety of mask manufacturers, small sample sizes for each mask and condition, and only 2 sterilization techniques evaluated. In addition, this study only compared the filtration efficiency after 1 sterilization cycle; however, filter material may degrade further after multiple cycles, which should also be investigated. To better protect health care personnel in hospitals, we recommend measuring the respirator’s filtration efficiency by aerosol size instead of only measuring the overall filtration efficiency.

## Notes

Supplement. **eFigure 1.** Experimental Setup for Mask Filtration Efficiency Test **eFigure 2.** Particle Size Distribution of Salt Testing Aerosol
