# Error in Product Description

**DOI:** 10.1001/jamanetworkopen.2020.18414

**Published:** 2020-08-14

In the research letter titled “Effects of Sterilization With Hydrogen Peroxide and Chlorine Dioxide Solution on the Filtration Efficiency of N95, KN95, and Surgical Face Masks,”^1^ published June 15, 2020, the disinfection method that used chlorine dioxide actually used a product called Vital Oxide, of which chlorine dioxide is the effective, but not only, ingredient. This article has been corrected.
