# Supplementary material for: Effects of Sterilization With Hydrogen Peroxide and Chlorine Dioxide on the Filtration Efficiency of N95, KN95, and Surgical Face Masks
Source: JAMA Netw Open. 2020 Jun 15;3(6):e2012099. doi: 10.1001/jamanetworkopen.2020.12099 (PMC7296389; doi:10.1001/jamanetworkopen.2020.12099)
Supplement: Supplement. — eFigure 1. Experimental Setup for Mask Filtration Efficiency Test eFigure 2. Particle Size Distribution of Salt Testing Aerosol [file jamanetwopen-3-e2012099-s001.pdf]

Supplementary Online Content

Cai C, Floyd EL. Effects of sterilization with hydrogen peroxide and chlorine dioxide on the filtration efficiency of N95, KN95, and surgical face masks. *JAMA Netw Open*. 2020;3(6):e2012099. doi:10.1001/jamanetworkopen.2020.12099

eFigure 1. Experimental Setup for Mask Filtration Efficiency Test

eFigure 2. Particle Size Distribution of Salt Testing Aerosol

This supplementary material has been provided by the authors to give readers additional information about their work.

© 2020 Cai C et al. *JAMA Network Open.*

eFigure 1. Experimental Setup for Mask Filtration Efficiency Test

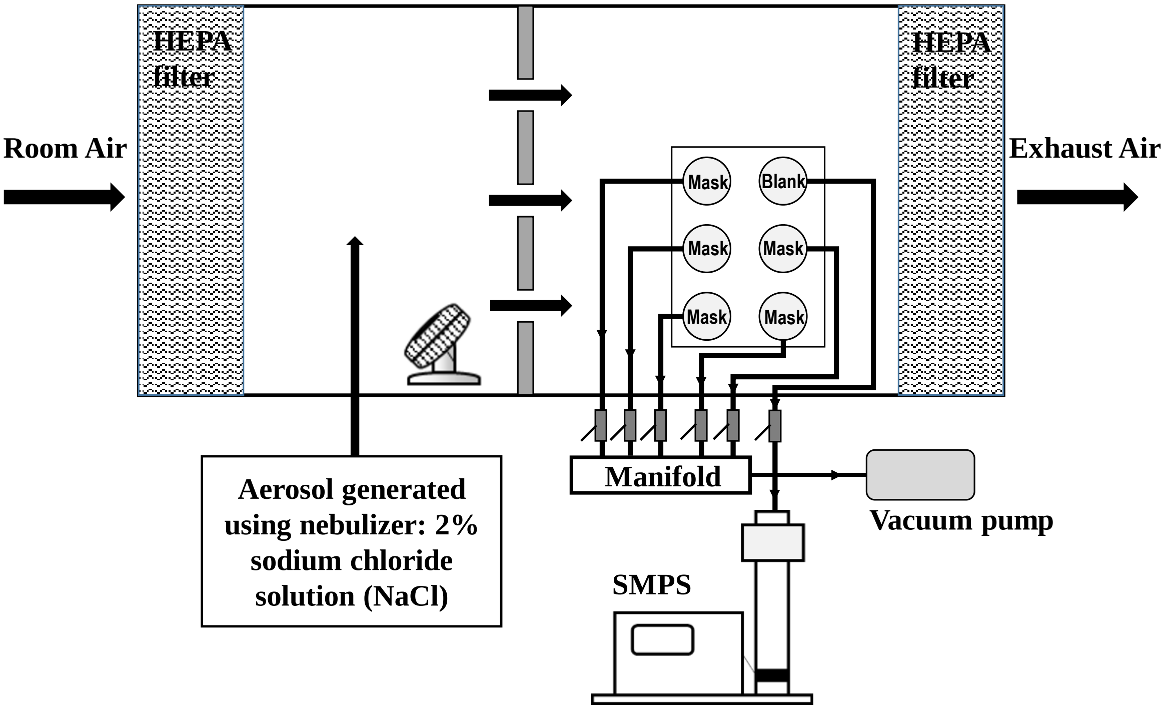

© 2020 Cai C et al. *JAMA Network Open.*

eFigure 2. Particle Size Distribution of Salt Testing Aerosol

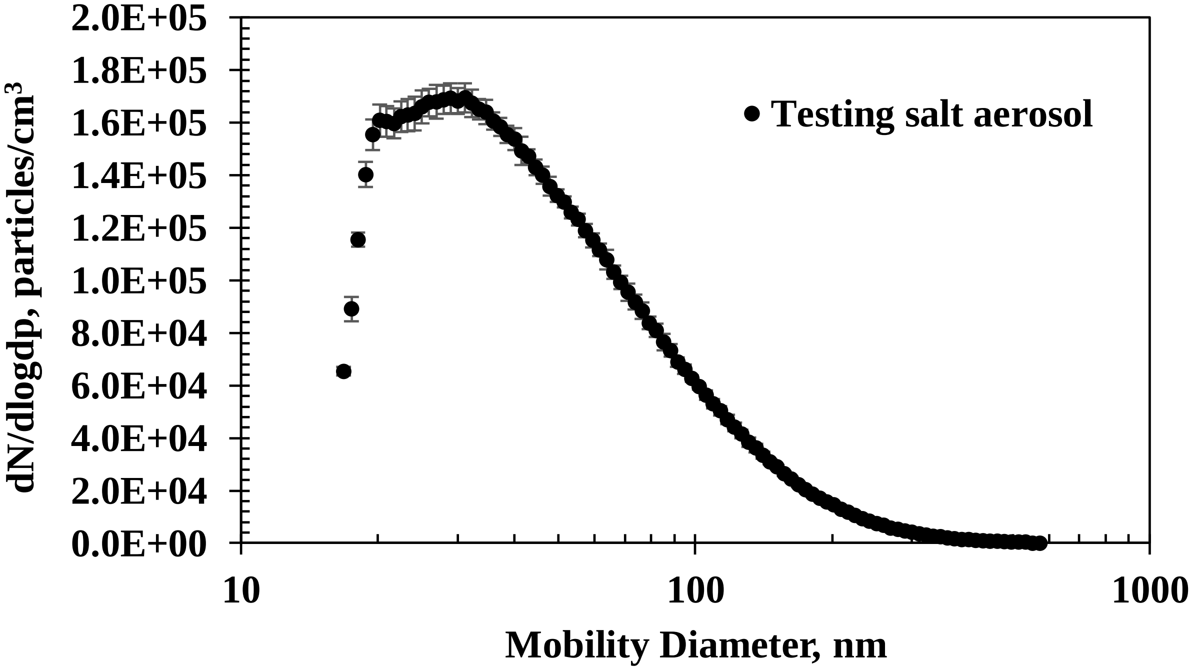

(Note: error bar denotes standard deviation.)

© 2020 Cai C et al. *JAMA Network Open.*
